# hiPSC-Derived Cardiac Tissue for Disease Modeling and Drug Discovery

**DOI:** 10.3390/ijms21238893

**Published:** 2020-11-24

**Authors:** Junjun Li, Ying Hua, Shigeru Miyagawa, Jingbo Zhang, Lingjun Li, Li Liu, Yoshiki Sawa

**Affiliations:** 1 Department of Cardiovascular Surgery, Osaka University Graduate School of Medicine, 2-2 Yamadaoka, Suita, Osaka 565-0871, Japan; jli@surg1.med.osaka-u.ac.jp (J.L.); y-hua@surg1.med.osaka-u.ac.jp (Y.H.); miyagawa@surg1.med.osaka-u.ac.jp (S.M.); jb-zhang@surg1.med.osaka-u.ac.jp (J.Z.); Lilingjun009@tissue.med.osaka-u.ac.jp (L.L.); 2 Department of Cell Design for Tissue Construction, Faculty of Medicine, Osaka University Graduate School of Medicine, 2-2 Yamadaoka, Suita, Osaka 565-0871, Japan; 3 Department of Design for Tissue Regeneration, Osaka University Graduate School of Medicine, 2-2 Yamadaoka, Suita, Osaka 565-0871, Japan

**Keywords:** hiPSC-derived cardiomyocytes, maturation, subtype, disease modeling, drug discovery

## Abstract

Relevant, predictive normal, or disease model systems are of vital importance for drug development. The difference between nonhuman models and humans could contribute to clinical trial failures despite ideal nonhuman results. As a potential substitute for animal models, human induced pluripotent stem cell (hiPSC)-derived cardiomyocytes (CMs) provide a powerful tool for drug toxicity screening, modeling cardiovascular diseases, and drug discovery. Here, we review recent hiPSC-CM disease models and discuss the features of hiPSC-CMs, including subtype and maturation and the tissue engineering technologies for drug assessment. Updates from the international multisite collaborators/administrations for development of novel drug discovery paradigms are also summarized.

## 1. Introduction

**Figure 1:**
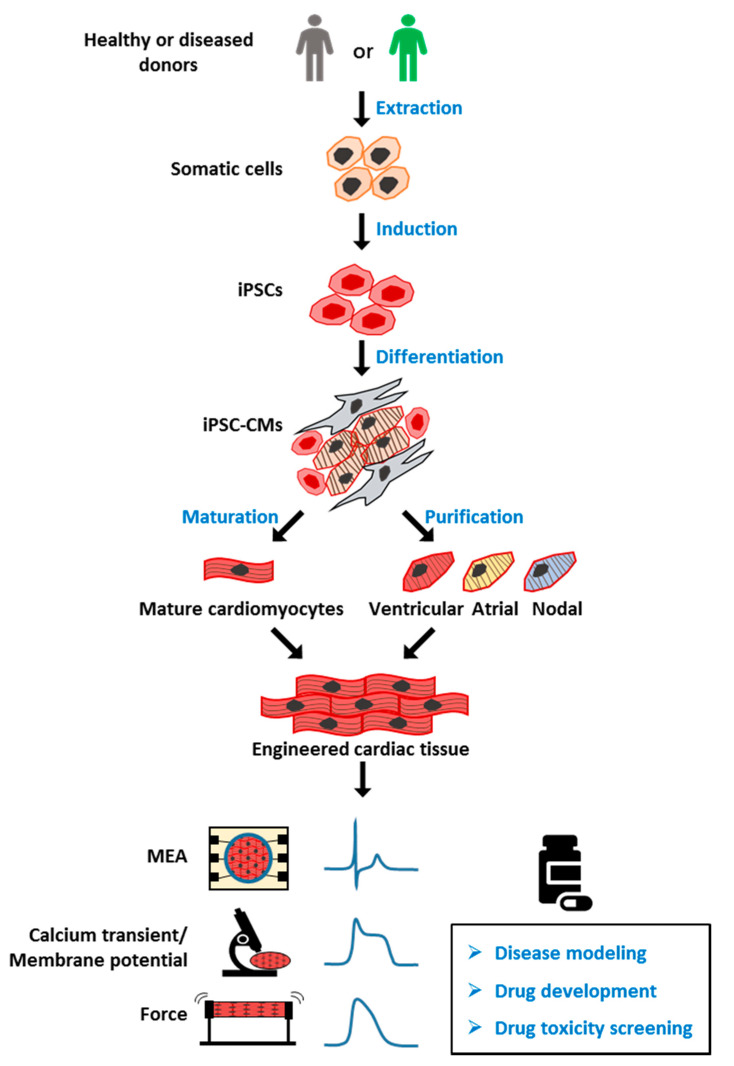
Overview of human induced pluripotent stem cell-derived cardiomyocyte (hiPSC-CM) model for drug screening. Healthy or patient-derived somatic cells can be reprogrammed into human induced pluripotent stem cells (hiPSCs) and then differentiated into all subtypes of cardiomyocytes (hiPSC-CMs), including ventricular, atrial, and nodal myocytes. iPSC-CMs can be matured and engineered into three-dimensional (3D) cardiac tissue, and used for applications including disease modeling, drug development, and toxicity screening. MEA: multielectrode array, for detection of extracellular field potential (FP) of CMs. Calcium transient: the intracellular calcium concentration during the CMs beating. Membrane potential: difference in electric potential between the inside and outside of cell membrane. The recording of membrane potential of CMs can be used for analyzing action potential (AP). Force: also known as contractile force, generated by the shift of the sarcomere. The contractile force and frequency are closely related to cell function.

Human induced pluripotent stem cells (hiPSCs) were developed by Dr. Shinya Yamanaka more than 10 years ago [1]. This technology allows pluripotent stem cells to be derived from healthy persons, as well as patients. hiPSCs have been used in multiple fields, leading to significant technological and therapeutic developments. hiPSC-derived cardiomyocytes (CMs) have been used to model several major cardiomyopathies, including ion related, structural, and metabolic cardiomyopathy, providing new insights into the mechanism underlying the disease phenotype. A potential genetic therapy based on CRISPR/Cas9 and adeno-associated virus has also been proposed and validated in an hiPSC disease model. Another promising application of hiPSC-CMs is drug toxicity screening (Figure 1); despite the remaining issues such as immaturity and heterogeneity within the hiPSC-derived CM culture, a new paradigm based on hiPSC-CMs has been proposed for more accurate prediction of the proarrhythmia risk.

In this review, we provide an overview of hiPSC-CMs and their features, including characterization, maturation, and tissue engineering. Their applications in cardiac disease modeling and new drug testing paradigms are also summarized and discussed.

## 2. Generation of Human iPSC-CMs and Their Subtypes

Cardiovascular disease (CVD) is a leading cause of the global deaths [2]. Modeling CVD is essential for understanding its causes and the therapies of such diseases. There are already reports on the use of human primary CMs to model human heart [3,4,5,6]; however, limited access to human samples and the variability of human material cause problems, since each tissue source can only be assessed once [3]. hiPSC-CMs can be obtained in large amounts, and they recapitulate the properties of human heart cells. Ventricular CMs, a chamber-specific CM population, have been differentiated with high purity and widely used in the study of drug responses and disease modeling. Notably, a chemically defined cardiac differentiation protocol was recently developed to produce ventricular-like CMs with >90% purity and on a large scale [7]. The heart is composed of multiple cell subtypes, including not only ventricular CMs, but also pacemaker cells and atrial myocytes [8]. These subtypes are all important to the proper functioning of heart. In order to obtain more accurate drug responses and better therapeutic effects, it is of vital importance to acquire tissue-specific cells and promote their maturation [9,10]. While significant progress has been achieved in ventricular tissue engineering, iPSC-derived atrial tissues are still immature.

In contrast to ventricular cardiomyocytes, atrial cells are smaller and thinner, and they have fewer transverse tubules (T-tubules) and less calcium-handling machinery. Retinoic acid (RA) was recently used to differentiate iPSCs into atrial CMs. Lee et al. developed an improved differentiation protocol for the generation of atrial linages by utilizing developmental signaling gradients that specify atrial mesoderm precursors [11]. More recently, by using a stage-specific activation of RA signaling in monolayer-based culture, Cyganek et al. demonstrated that cardiac progenitors could be efficiently directed toward a highly homogeneous population of atrial CMs [12]. Zhao et al. described a scalable tissue-cultivation platform that can electrophysiologically distinguish atrial and ventricular tissues with chamber-specific drug responses and gene expression [13]. These studies provide a solid foundation for further generation of atrial tissues.

Pacemakers are important for patients suffering from cardiac arrhythmia. There are numerous reports on using different protocols to obtain and evaluate cell populations from the cardiac conduction system. White et al. successfully developed a cardiac pacemaking conduction system via co-expression of the chicken GATA6 enhancer and mink-lacZ transgene [14]. Yano et al. also reported that a fraction of Nkx2.5-positive cardiac precursor cells were committed to pacemaker cells expressing *I*_f_ channels predominantly encoded by the hyperpolarization-activated cyclic nucleotide-gated 1 (HCN1) and HCN4 genes [15]. It was reported that the addition of B12 or SKCa activator during differentiation can promote an increase in the nodal population [16]. Moreover, via stage-specific manipulation of developmental signaling pathways, Protze et al. developed a transgene-independent protocol to differentiate sinoatrial node cells from iPSCs [17]. Further efforts are needed to develop a robust differentiation protocol for generating mature ventricular CMs, atrial CMs, and pacemakers to enable better myocardium recapitulation.

## 3. Patient-Specific iPSC-CMs as Disease Models

iPSCs have been derived from patients and introduced into various patient-specific iPSC-CMs for modeling cardiomyopathies in vitro. These models can be categorized as inherited or nonhereditary, and they are reviewed below (Table 1).

### 3.1. Inherited Cardiomyopathy

#### 3.1.1. Ion Channelopathy

Ion channelopathies are one of the most well-established iPSC-based disease models because of their better-understood impact on action potential (AP) [18]. Abnormalities occurring in AP generation, synchronization, or propagation may cause cardiac channelopathies related to arrhythmia [19]. The most common ion channelopathy is long QT syndrome (LQTS), which has a prevalence of 1 in 2000 [20]. Decreased systolic Ca^2+^ release leads to impaired cellular contractility and delayed repolarization of ventricular CMs. LQTS is characterized by a prolonged QT interval, causing increased risk of ventricular tachyarrhythmia or sudden cardiac death [21]. LQTS is divided into more than 10 different subtypes defined by specific ion channel mutations. Among these, the voltage-gated sodium (Na_v_) channel and the cardiac voltage-gated potassium (K_v_) channel, both electrical impulse-initiating ion channels, are the primary mutation types [22]. LQTS type 1 (LQT1) occurs due to mutations in *KCNQ1*, and LQT1 iPSC-CM disease modeling was first generated by Moretti and his collages [23]. Different disease-specific human iPSC lines have been developed from patients with these ion channel gene mutations. The LQT1 disease model accurately reflects the disease features, having a slow outward potassium current (*I*_Ks_), abnormal channel activities, and increased susceptibility to tachyarrhythmia induced by catecholamine [24,25]. LQT2 has mutations in *KCNH2*, a human ether-à-go-go-related gene (hERG) that mediates rapid delayed-rectifier potassium current *I*_Kr_, which is important for the repolarization phase of the AP [26]. Thus, the LQT2 disease models revealed significant prolongation of the action potential duration (APD) and a reduction in *I*_Kr_ when compared to healthy control cells [27]. Precise genetic modification of the *KCNH2* mutation increased the *I*_Kr_ current conducted by the hERG channel and normalized the APD [28]. Malan et al. developed hiPSC-CMs from an LQT3 patient with an *SCN5A* mutation, known to mediate fast Na_v_1.5 channel inactivation. LQT3 hiPSC-CMs exhibited accelerated recovery from Na_v_1.5 inactivation, AP prolongation, and early afterdepolarizations (EADs) even at low stimulation rates, which are considered to be the main cause of arrhythmia [29]. Roche et al. investigated the *SCN5A* mutation, using different independent systems, and compared the advantages and limitations of disease-specific, engineered iPSC-CMs and heterologous HEK293-cells for disease modeling and drug discovery, emphasizing the importance of investigating the mechanisms of Brugada syndrome in independent systems [30]. These cell-based models indicated that ion-trafficking defects are the associated pathological mechanism of the disease electrophysiological phenotype, and that regulation of key genes may be governed by a complex regulatory landscape.

Another common inherited channelopathy is catecholaminergic polymorphic ventricular tachycardia (CPVT), mainly caused by mutations in calcium-handling genes characterized by Ca^2+^ cycling and electrophysiology defects in patients [31]. The most prevalent CPVT1, responsible for 60% of total cases, is caused by mutations in *RYR2*, which encodes the cardiac ryanodine receptor. CPVT2 is less common, causing less than 5% of total cases, and is produced by a mutation in *CASQ2*, encoding cardiac calsequestrin [32]. Both mutations lead to abnormal calcium leakage from the sarcoplasmic reticulum (SR), causing cytosolic calcium overload and subsequent delayed afterdepolarizations and triggering ventricular arrhythmias [33]. CPVT patient-derived hiPSC-CMs carrying either *RYR2* or *CASQ2* mutations have been generated by several groups. Using selective pharmacology and genome editing, Park et al. generated a novel model that effectively recapitulates the CPVT1 profile caused by dominant mutations in *RYR2* [34]. They regarded the activation of Ca^2+^/calmodulin-dependent protein kinase (CaMK II) as a key factor for triggering arrhythmias in CPVT patients, suggesting a molecular pathway linking β-adrenergic stimulation to arrhythmogenesis. Using these disease models, a series of potential compounds have been tested for modifying aberrant Ca^2+^ handling and delayed afterdepolarizations (DADs) [35,36,37,38].

#### 3.1.2. Structural Cardiomyopathy

Hypertrophic cardiomyopathy (HCM) is a common inherited heart disease with abnormalities in morphology, with an estimated prevalence of 1 in 500 worldwide [39]. Most cases of sudden death related to HCM are caused by the conversion of ventricular arrhythmia to ventricular fibrillation [40]. Over 1500 mutations have been identified in HCM, most of which are located in sarcomere genes. These genes are responsible for CM contraction and relaxation [41]. Approximately 70% of HCM patients had either *MYH7* (encoding β-myosin heavy chain) or *MYBPC3* (myosin-binding protein) mutations, and their heart explants revealed lower tension forces compared to healthy individuals [42]. Less common mutations are located in other sarcomere genes such as actin (*ACTC*), cardiac troponin T (*TNNT2*), myosin light chain (*MYL2*), and cardiac troponin I (*TNNI3*) or non-sarcomere genes such as ion channels, Z-disc genes, and membrane transporters [41]. Five HCM hiPSC-CM models have been derived from patients carrying either *MHY7* or *MYBPC3* mutations using viral vectors [43]. Intraventricular injection of adeno-associated virus has shown potential as therapy for treating the MYL2 mutation in heart cells [44]. CRISPR/Cas9 editing has also been used to generate HCM disease modeling with site-directed homozygous or heterozygous variants [45]. These cell-based models recapitulate key features of the HCM phenotype such as increased sarcomere organization and aberrant Ca^2+^ handling, providing a new in vitro model for identifying pathogenesis and developing new therapeutic strategies for these inherited heart disease [46]. However, engineered heart tissue (EHT) has more advantages in terms of mechanism elucidation than single-cell models, as EHT can better reflect and mimic the cell–cell interaction at the tissue level. Cashman et al. first developed EHTs created from cardio-facio-cutaneous syndrome (CFCS) patients with *BRAF* mutations (encoding a serine/threonine kinase), and this tissue-based model better recapitulated key aspects of the HCM phenotype in vivo, providing a powerful tool for studying the patient-specific mechanisms of myocardial dysfunction [47].

Dilated cardiomyopathy (DCM) is another type of structural cardiomyopathy. DCM is mainly due to sarcomere mutations and has a prevalence of 1 in 2500 individuals [48]. Hearts affected by DCM tend to have increased chamber size and thinner chamber walls leading to volume overload, systolic dysfunction, and progressive heart failure (HF) [49]. DCM has high morbidity and mortality rates and is the leading cause of HF in young people. More than 80 different genes associated with DCM have been described. Of these, *TTN* encoding titin is the most prevalent mutant gene identified in around 20–25% of DCM patients [50]. Sun et al. first developed DCM iPSC-CMs with a mutation in *TNNT2*, recapitulating the DCM disease phenotypes morphologically and functionally. Their model has altered Ca^2+^ handling, decreased contractility, and abnormal α-actin distribution [51]. Dai et al. revealed that the *TNNT* mutation destabilizes the molecular interactions of troponin with tropomyosin and limits PKA binding to sarcomere [52]. Mutations occur less commonly in nuclear lamina, Na_v_ channel α-subunit 5 (*SCN5A*), desmin (*DES*), phospholamban (*PLN*), Bcl2-associated athanogene 3 (*BAG3*), and RNA-binding motif protein 20 [53].

Arrhythmogenic cardiomyopathy (ACM) is another common structural cardiomyopathy usually caused by mutations in desmosomal proteins, leading to progressive HF and lethal arrhythmias [54]. Approximately half of the patients with ACM have gene mutations in desmosomes, such as desmoplakin (*DSP*), desmocollin (*DSC*), desmoglein-2 (*DSG2*), plakoglobin (*JUP*), and plakophilin-2 (*PKP2*). The *PKP2* mutation is the most common pathogenic type in ACM [54]. hiPSC-CMs with a *PKP2* mutation recapitulated key features of arrhythmogenic right-ventricular cardiomyopathy (ARVC), including low β-catenin activity, abnormal nuclear translocation of junction plakoglobin, and less cell surface localization of desmosomes, presenting an adipogenic phenotype [55]. However, it was reported by Kim et al. that only by co-activating peroxisome proliferator-activated receptor (PPAR)-α/PPAR-γ pathways, both of which are responsible for metabolism, can iPSC-CMs with a *PKP2* mutation display efficient ACM features within 2 months [56,57]. This report proposed for the first time that induction of adult-like metabolism phenotype plays a role in adult-onset disease modeling.

Duchenne muscular dystrophy (DMD) is a rare X-linked recessive disease with an incidence of 1 per 5000 males. The cells of DMD patient are highly susceptible to mechanical stress and injury as they lack the dystrophin protein [58]. Dystrophin is a fundamental component of the dystrophin–glycoprotein complex, which is expressed at the muscle sarcolemma and bridges the cytoskeleton and extracellular matrix, maintaining cellular stability [59]. Dystrophin deficiency leads to progressive muscle scarring and degeneration, HF, and eventually death. DMD patient-derived iPSC-CMs exhibited excessive Ca^2+^ influx and increased sensitivity to hypotonic stress, accumulation of reactive oxygen species (ROS), and mitochondrial damage, eventually inducing cell apoptosis [60]. Dystrophin modifications by CRISPR/Cas9 have been proven to be a fast way to rescue DMD, with efficient restoration of CM contractility and calcium transients detected to varying degrees [61].

#### 3.1.3. Metabolic Cardiomyopathy

Acid-α-glucosidase (GAA) is an amylolytic enzyme located in the lysosome and is responsible for glycogen degradation. Deficiency of GAA results in the accumulation of glycogen in lysosomes, a condition called Pompe disease (PD) [62]. As a result of dysregulation of glycogen metabolism, PD myocytes display increased cytoplasmic glycogen particles, endoplasmic reticulum stress, mitochondrial aberrance, abnormal calcium signaling, and progressive autophagic buildup [63]. The PD can be divided into infantile and late-onset phenotypes. Huang et al. reported that derivation of infantile-onset PD-iPSCs into CM-like cells recapitulated the hallmark of PD cells, including glycogen accumulation and differential ultrastructural aberrations [64]. A subsequent drug rescue test showed that GAA or I-carnitine could reverse the major pathologic phenotypes. Raval et al. investigated the mechanism of PD in tissue using a generated EHT model [65]. They stated that the lack of GAA ability leads to deficits in Golgi-based protein glycosylation, thus finally leading to lysosomal glycogen accumulation and HCM. Sato et al. generated late-onset Pompe disease-specific iPSC-CMs and showed that glycogen accumulation can be ameliorated by lentiviral GAA rescue [66]. Using metabolic profile analysis, they found that oxidative stress and mitochondrial dysfunction induced in the PD model may be related to cardiac complications [67]. The imbalance between oxidative stress and an antioxidative stress response may, therefore, reveal the pathogenesis of late-onset PD.

Barth syndrome (BTHS) is an X-linked mitochondrial disorder caused by a mutation in tafazzin, an acyltransferase encoded by *TAZ* [68]. Tafazzin is responsible for the normal acylation of cardiolipin, which is mainly located in the mitochondrial inner membrane. BTHS features multisystem disorders such as cardiomyopathy, neutropenia, and skeletal myopathy [69]. Using BTHS patient-derived iPSC-CMs, Wang et al. investigated the structural, metabolic, and functional abnormalities caused by *TAZ* mutation [70]. They engineered BTHS iPSC-CMs into a “heart-on-chip” and demonstrated sparse and irregular sarcomeres with weak contractile force in this chip. These findings indicate the presence of a link between *TAZ* mutation and impaired CM mechanical function, providing new insights into the pathogenesis of BTHS.

### 3.2. Chronic Nonhereditary Cardiomyopathy

Chronic heart failure (CHF), such as congestive heart failure, is a progressive syndrome caused by CVDs including coronary artery disease and myocardial infarction, as well as high blood pressure, and it results in structural or functional changes in the heart [71]. Heart failure with reduced ejection fraction (HFrEF) is a common type of CHF, usually caused by long-term use of catecholamines (e.g., norepinephrine) in patients with end-stage HF [72]. HFrEF hearts show hypertrophy, a weaker force–frequency response, and decreased β-adrenergic sensitization [73]. Through chronic norepinephrine stimulation, Tiburcy et al. successfully generated HF models with not only pathological hypertrophy, cellular death, and contractile dysfunction, but also N-terminal pro B-type natriuretic peptide (NT-proBNP) release, features consistent with the clinical diagnosis of HF [74]. Their work provides guidance for the establishment of HF modeling, drug screening, and tissue-based heart repair.

**Table 1:** Categories of patient-specific iPSC-CMs as disease models.

Disease Model Categories				Related Genes	Reference
Inherited cardiomyopathy	Ion Channelopathy	Long QT syndrome (LQTS)	Type 1	*KCNQ1*	[23,24,25]
			Type 2	*KCNH2*	[27,28]
			Type 3	*SCN5A*	[29,30]
		Catecholaminergic polymorphic ventricular tachycardia (CPVT)		*RYR2*	[34]
	Structural Cardiomyopathy	Hypertrophic cardiomyopathy (HCM)		*MYH7, MYBPC3, ACTC, TNNT2, MYL2, TNNI3*	[43,45,47]
		Dilated cardiomyopathy (DCM)		*TTN, TNNT2, SCN5A, DES, PLN, BAG3*	[51,52,53]
		Arrhythmogenic cardiomyopathy (ACM)		*DSP, DSC, DSG2 JUP, PKP2*	[54,56]
		Duchenne muscular dystrophy (DMD)		*DMD*	[60]
	Metabolic cardiomyopathy	Pompe disease (PD)		*GAA*	[64,65,66]
		Barth syndrome (BTHS)		*TAZ*	[70]
Chronic nonhereditary cardiomyopaty	Chronic Heart Failure	Heart failure with reduced ejection fraction (HFrEF)		N/A	[74]

## 4. Maturation Differences between hiPSC-CMs and Adult CMs

Although multiple subtypes of CMs have been induced from either healthy donors or patients for applications such as drug screening, disease models, and regenerative medicine, they still remain immature in terms of microstructure, electromechanics, and metabolism compared with adult CMs. This shortcoming limits the application of hiPSC-CMs. We summarized the gap between hiPSC-CMs and adult CMs in these aspects (Table 2).

### 4.1. Morphology and Structure

Human cardiac myocytes exit the cell cycle and become quiescent with further growth in size and increased maturation in the first decade after birth [75,76]. Adult CMs always appear polyploidy with an elongated, anisotropic, and well-developed SR, T-tubules, mitochondria, highly organized sarcomere structures, and polarized intercalated disc (ID) complexes including desmosomes, gap junctions (GJs), and adhesive junctions (AJs) [77,78]. Sarcomeres are longitudinally repeated subunits of myofibrils that serve as the contractile apparatus of CMs. However, newly differentiated hiPSC-CMs tend to be monoploid and have poor myofibrils, as well as other essential ultrastructures, in addition to a small, rounded shape [79]. Moreover, the isoforms and expression of myofibril assembly-related proteins in hiPSC-CMs, such as α-actin, titin, myosin heavy chain (MHC), myosin regulatory light chain 2 (MLC2), and the troponin complex, are also less mature than their adult counterparts [80,81]. Although the cellular structure was compatible with this function, the deficiency of key substructures resulted in poor ion activities, slower Ca^2+^ handling, and inefficient mitochondrial metabolism in hiPSC-CMs.

### 4.2. Electrophysiological Properties

The electrophysiological phenotype of hiPSC-CMs is distinct from that of adult CMs, mainly due to the differential expression of key ion channels and GJ proteins. hiPSC-CMs beat spontaneously because of the high expression of potassium hyperpolarization-activated cyclic nucleotide-gated channel 4 (HCN4, encoded by *HCN4*), which is related to sodium influx in the pacemaker, while adult CMs only beat when stimulated with a 40–80 mN/mm^2^ force [82]. The resting membrane potential of hiPSC-CMs is less negative (~−60 mV) than mature CMs (~−90 mV) as a result of insufficient expression of Kir2.1 (encoded by *KCNJ2*) [83]. Furthermore, the upstroke velocity of hiPSC-CMs (~15–30 V/s) is much slower due to lacking sodium channels such as Na_v_1.5 (encoded by *SCN5A*) and lower membrane capacitance (~20 pF) related to the smaller size than that of mature CMs with fast upstroke velocity (~−150–350 V/s) and higher capacitance (~190 pF) [84]. hiPSC-CMs also have a shorter plateau phase, partly due to the low expression of voltage-gated L-type calcium channel (LTCC) alpha subunit (Ca_v_1.2), which mediates influx of Ca^2+^ into the cytoplasm [85,86]. Lastly, the electrical conduction speed in hiPSC-CMs (~10 cm/s) tends to be much lower than that in adult CMs (~100 cm/s) due to the lower expression of GJs and their circumferential distribution in hiPSC-CMs [87].

### 4.3. Calcium Handling

Excitation–contraction coupling (ECC) is the foundation of muscular activity. In ECC, the opening of LTCC caused by APD leads to an influx of extracellular Ca^2+^ and subsequent release of SR Ca^2+^ through ryanodine receptors (RyRs), causing Ca^2+^ accumulation in the cytoplasm [88]. Upon muscle contraction, Ca^2+^ binds to troponin C and triggers myofilament displacement, and contraction occurs. Then, cytoplasmic Ca^2+^ flows out of the cell via the Na^+^–Ca^2+^ exchanger (NCX) or back to the SR (SR/ER intracellular calcium pool) through the sarco/endoplasmic reticulum Ca^2+^ ATPase (SERCA), and relaxation finishes [88]. In adult CMs, T-tubules are an invagination of the cytoplasmic membrane, and such specialized structures cause spatial coupling of LTCC and RyRs [89]. However, the lack of T-tubules, undeveloped SR, and lower expression of key calcium-handling proteins in hiPSC-CMs, such as SERCA, calsequestrin (calcium-buffering protein), and Ca_v_β2 (LTCC β-subunit), slow calcium dynamics with weak Ca^2+^ signal and peak delay [90,91].

### 4.4. Metabolism

Metabolic transition occurs swiftly after birth, due to the significant increase in oxygen and nutrients in the blood. Mitochondria continuously undergo morphological and physiological changes during CM maturation for a decade after birth [92]. In adult CMs, mitochondria with developed cristae and oval shape are well organized, located among myofibrils and under the sarcolemma, making up ~30% of total cell volume [93]. More than 95% of the total ATP consumed in adult CMs is supplied by fatty-acid β-oxidation (FAO) in the mitochondria; two-thirds of the generated ATP supports contraction, whereas the remaining one-third is used for ion pumps [94]. However, hiPSC-CMs share the same mitochondrial phenotype as fetal CMs, located around the nucleus with smaller size, fewer numbers (<5% of cell volume), and poor cristae. More than 80% of the energy is generated from glycolysis rather than FAO. In addition, low fatty-acid utilization is partly due to low transport efficiency of fatty acids and low expression of electron-transport-chain proteins.

## 5. Approaches for CM Maturation

### 5.1. Prolonged Culture Time

It takes human CMs a decade to reach full maturation in vivo [78]. When cultured for a year, hiPSC-CMs were larger in size and had aligned sarcomeres, improved calcium handling, and other key features as observed in adult CMs, such as M-bands, indicating that the hiPSC-CMs had a more mature phenotype [95,96] (Table 3). Although long-term culture does facilitate the maturation of hiPSC-CM in various aspects, it is time-consuming and costly, and it is unable to induce T-tubules; thus, it may not meet the requirements of drug screening and development [74].

### 5.2. Biochemical Cues

Cardiovascular homeostasis plays an important role in heart development by producing a dynamic microenvironment, a synergistic effect of hormones and cytokines. The levels of thyroid hormones and endogenous glucocorticoids (GCs) rise sharply around the time of birth, and it has been reported that both are essential for normal heart maturation [97,98]. Treatment with tri-iodothyronine (T3) strongly accelerates contractile force and metabolism maturation, and induces cell-cycle arrest of hiPSC-CMs in vitro [99]. A combination of T3 and GCs or dexamethasone, an analogue of GCs, can synergistically induce further maturation of hiPSC-CMs in vitro, including the formation of T-tubules, which cannot be achieved by any single drug [100]. Moreover, combined T3 and GCs or its analogue, dexamethasone, can synergistically induce the formation of T-tubules and improve calcium kinetics in hiPSC-CMs [101]. Angiotensin II, a main effector peptide, functions in the regulation of blood pressure in the renin–angiotensin system, and it can also promote human embryonic stem cell (hESC)-CM hypertrophy by activating the MAPK signaling pathway, which is involved in sarcomeric organization during CM differentiation [102].

Cytokine factors act via autocrine and paracrine effects. Normal cardiac metabolism requires insulin-like growth factor (IGF) and neuregulin-1 (NRG1), both independently and synergistically [103,104]. Zhou et al. demonstrated that IGF acts as an activator of Akt, thereby prompting reprogramming of fibroblasts to functional CMs [105]. These cells are polynucleated and exhibit cellular hypertrophy and enhanced mitochondrial respiration. NRG1 enhances the patterning of the ventricular trabecule layer and endocardial cushion, both crucial for formation of heart chambers [106]. When treated with NRG1 alone, hESC-CMs matured with cell growth and metabolic remodeling. The synergistic force–frequency relationship was improved upon treatment with NRG1 combined with IGF [107]. Other cytokines, such as fibroblast growth factor (FGF), transforming growth factor beta (TGF-β), and vascular endothelial growth factor (VEGF), play roles in the maintenance of GJs and aggregation in the three-dimensional (3D) culture system of hPSC-CMs [108,109,110].

A significant postnatal CM maturation is the metabolic switch from glycolysis to oxidative phosphorylation (OXPHOS). Correia et al. reported that the medium in which glucose is replaced with galactose and fatty acids facilitates the overall maturation of iPSC-CMs into adult-like CMs [111]. Yang et al. also verified that switching the energy source affects mitobiogenesis and FAO in iPSC-CMs. Moreover, galactose plays a role in ameliorating lipotoxicity resulting from high fatty-acid exposure [112]. The studies have bridged substrate utilization and functional maturation of hPSC-CMs and will facilitate the application of iPSC-CMs in clinical and preclinical studies.

Oxygenation level is a critical cue in CM differentiation, suggesting that hypoxia may play a negative role in early cardiogenesis [113]. Hypoxia-inducible factor 1-alpha (HIF-1α), a central regulator of metabolism, can be upregulated by hypoxia, whereas increased oxygen tension inhibits HIF-1α activity and promotes the metabolic switch to OXPHOS during murine heart development [114]. However, the high-glucose culture medium, which is widely used for the culture of hiPSC-CMs, activates HIF-1α and upregulates lactate dehydrogenase A (LDHA), thereby facilitating glycolysis while suppressing OXPHOS; this is considered to hinder cell metabolic maturation [115]. When the HIF-1α–LDHA axis is suppressed by chemical or small interfering RNA (siRNA) inhibition, hiPSC-CM metabolism swiftly shifts from anaerobic glycolysis to OXPHOS and shows enhanced maturation [116]. These findings provide key insights into the molecular control of hPSC-CM metabolism during maturation.

### 5.3. Biophysical Cues

Mechanical and electrical stimulation are widely used to promote maturation of hiPSC-CMs in vitro. Both neonatal and adult CMs are assumed to have a rod-like and elongated shape with an average length-to-width ratio of 7:1 in vivo, but revert to a rounded shape after being cultured in a static standard culture condition [117]. It is considered that the standard culture conditions do not exhibit the hemodynamic environment which is present in vivo. Either electrical or mechanical stimulation could lead to structural maturation of hiPSC-CMs with rod-like morphology and more aligned sarcomeres [118,119,120]. After exposure to synchronized mechanical and electrical stimulation, hiPSC-CMs displayed enhanced structural maturity and positive-ion activities. This activity is partially due to the enhancement and polarization of GJs promoting electrical conduction [121].

Cellular interactions should be taken into account while considering CM maturation. In vivo, extracellular matrices (ECM), which are mainly produced by endothelial cells and fibroblasts, are abundant, facilitate normal CM shape, and participate in mechanotransduction pathways [122]. In an in vitro hiPSC-CM culture system, not only natural animal ECMs but also artificial polymers, especially polydimethylsiloxane (PDMS), are often used to create scaffolds to mimic the ECM crosslink [123,124]. Xu et al. presented a onion epithelium-like biomimetic microchip made from PDMS to promote in vitro cellular organization [125]. This microchip technology presents new insights into hiPSC-CM maturation enhancement by biophysical factors. Lyra-Leite et al. reported that mitochondrial function can also be regulated by ECM elasticity, whereas mitochondrial stress responses are regulated by both matrix elasticity and tissue alignment [126]. The Ca^2+^ handling of iPSC-CMs cultured on PDMS substrates was significantly enhanced with a faster upstroke velocity and improved SR Ca^2+^ cycling, while these alterations were independent of gene expression [127].

### 5.4. Co-Culture

The heart grows in a multicellular environment and noncardiomyocytes (non-CMs), such as endothelial cells, cardiac fibroblasts and leukocytes, occupy around 15–30% in volume of the mammalian fetal heart and maintain sustainable proliferation during cardiomyocyte development [128]. ECM is mainly produced by non-CMs and provides mechanical support for cardiomyocytes; non-CMs secrete cytokines to facilitate CM development through paracrine effects. Yoshida et al. co-cultured hiPSC-CMs in vitro with non-CMs including mesenchymal stem cells (MSCs) and endothelial cells (ECs), resulting in improved maturation of hiPSC-CMs [80]. When hiPSC-CMs were cultured only with soluble factors containing cytokines and exosomes secreted by MSCs, the iPSC-CMs also exhibited enhanced maturation, revealing paracrine effects of co-cultured non-CMs. In addition to types of non-CMs, the proportion of non-CMs is also critical for generating functional iPSC-CMs in an in vitro co-culture system. Iseoka et al. reported that, when co-cultured with non-CMs, iPSC-CMs occupied 30–50% of total cells, exhibiting stable structures, and they had increased cardiotherapeutic potential compared with other ratios [129].

### 5.5. 3D Cardiac Tissues

Conventional two-dimensional (2D) culture systems (monolayer hiPSC-CMs) are popular because of their simplicity and moderate scalability [130]. However, 2D cultures fail to recapitulate in vivo conditions, such as cellular crosstalk, tissue architectures, and extracellular microenvironments [131]. On the other hand, 3D culture systems could include cellular elements, ECM scaffolds, and fluidic microenvironments, which could be an ideal tool to mimic the cell–cell interaction in vivo [132]. Therefore, engineered heart tissues/muscle (EHT/EHM) has advantages in terms of mechanism elucidation and reconstitution of adult myocardium at the tissue level.

Zimmermann et al. first developed adult-like 3D-EHTs with neonatal rat cardiac myocytes and ECM proteins, representing a new approach to in vitro cardiac function research and heart repair based on ETH [133]. Later studies showed that hiPSC-CMs EHT could be matured by using electrical and mechanical stimulation or passive afterload. Hirt et al. described that continuous electrical stimulation induced further maturation in both rat EHT and human ETH with a denser cellular network, well-developed ultrastructure including M-bands and GJs, and increased ion activities [134]. By concurrent electromechanical stimulation at physiological frequency, Godier-Furnémont et al. observed a positive force–frequency relationship (FFR) for the first time in mammalian EHM, denoting functional maturation of EHM associated with increased calcium handling [135]. Leonard et al. verified that a suitable afterload is beneficial for functional maturation of hiPSC-CMs in EHTs [136].

More recently, it was reported that EHT could promote maturation close to the adult level by applying rapid electrical stimulation in a specific time window. Takeda et al. utilized a newly developed 3D artificial tissue by coating ECMs on single-cell surfaces for cardiotoxicity assays [137]. These 3D-hiPSC-heart tissues are useful for drug screening or cardiotoxicity assays, as the system showed doxorubicin sensitivity and hERG channel blocking profile in vitro. The 3D structure facilitates CMs self-organizing into an advanced and complex structure. Li et al. developed a device based on a low-attachment substrate where hiPSC-CMs can self-organize into a 3D tissue ring [138,139]. Without any external stimulation, the tissue ring cultured in the 3D system could spontaneously generate re-entrant waves, accompanied by rapid pacing, thus leading to enhanced maturation of CMs in an autonomous manner.

As mentioned above, ECMs are mainly produced by non-CMs and for better heart tissue organization; thus, non-CMs are also needed for ECM remodeling in the process from hiPSC-CMs to EHT. Non-CMs, such as endothelial cells, cardiac fibroblasts, and leukocytes, occupy ~15-30% of the total cardiac volume in mammalian fetal heart and retain sustainable proliferation during CM development [95]. The quantity and type of non-CMs are critical for generating functional iPSC-CMs in an in vitro co-culture system. ECTs containing 50-70% CMs combined with non-CMs exhibited stable structures and increased cardiotherapeutic potential [129].

Organoids, 3D cultures of multiple cell types, could partially recapitulate in vivo tissue or organ structure and function. Richards et al. reported a scaffold-free hiPSC-derived cardiac organoid that structurally and functionally resembles the vascular structure within the developing myocardium. This platform facilitates the investigation of cellular, matrix/material, and addition factors required for heart development [140]. More recently, the same group modeled the myocardial infarction by using cardiac organoids that recreate the necessary features, including fibrosis, metabolic shift, and pathological calcium-handling properties [141]. Buono et al. developed organoids derived from HCM patients. These organoids demonstrated significant phenotype of the hypertrophic cardiomyopathic human heart in comparison to the healthy control [142]. Monsanto et al. reported scaffold-free 3D organoids, termed CardioClusters, with controllable cell ratio and size, as well as minimal cell loss. The injection of these CardioClusters into a murine infarction model promoted the cell retention, capillary density, and heart function during the 20 week observation [143].

### 5.6. Regulation on the Molecular Level

Single-cell RNA sequencing studies indicated that gene expression patterns in adults are quite distinct from those in fetal heart and hiPSC-CMs; thus, regulation of intercellular gene expression has been one of the most popular methods for increasing hiPSC-CM maturation. Serum response factor (SRF), an important transcriptional regulator, impacts almost every aspect of CM maturation, partly due to its key role in regulating sarcomere genes [144]. SRF depletion significantly affect CM development, such as impaired sarcomeres, T-tubules, and mitochondria. Some major SRF coactivators, including myocardin family transcriptional regulator (MRTF), homeodomain-only protein homeobox (HOPX), GATA family transcription factor (GATA), myocyte enhancer factor-2 (MEF2), and nuclear receptor superfamily (NRs), mediate various progress in CM maturation according to different extracellular stimuli. The MRTF–SRF axis can convert mechanical stress into sarcomere expansion [145]. HOPX, a novel co-factor of SRF for CM maturation, functions in the process of myofibrillar isoform switching and CM hypertrophy with preserved systolic function [146,147].

SRF functions in synergy with GATA and MEF2 motifs in a maturing heart, although the role of the two is still controversial [148,149]. NRs are another major group of transcription regulators related to SRF that control CM maturation. Such factors include the heterodimers formed by peroxisome proliferator-activated receptors (PPARs)/retinoid X receptors (RXRs) and estrogen-related receptors α, β, and γ (ERRs) [150,151]. Both PPARs and ERRs directly interact with PGC1α/β (PPARγ coactivator α/β), the master regulators of FAO and mitochondrial respiration [152,153,154].

Epigenetic modifications exert a profound impact on transcriptional regulation, such as DNA methylation, histone modification and chromatin remodeling [155]. DNA hypermethylation is associated with gene silencing, whereas demethylation results in gene activation during CM maturation [156]. It has been reported that activation of H3K27ac, H3K4me1, H3K4me3, and H3K9ac is associated with increasing maturation in CMs [89,157].

MicroRNAs (miRNA) represent a posttranscriptional regulation method that modulates gene expression by silencing key messenger RNA (mRNA). miR-1 and Let-7i are highly enriched in mature CMs and these factors could facilitate electrophysiological maturation and respiratory capacity respectively when overexpressed in hiPSC-CMs [158,159]. Simultaneous overexpression of miR-125b-5p, miR-199a-5p, miR-221, and miR-222 resulted in improved maturation including α/β-MHC switching, sarcomere alignment, mitochondrial cristae formation, and improved Ca^2+^ handling [160]. Overexpressed Let-7i and miR-452 but repressed miR-122 and miR-200a were shown to be a new in vitro maturation cocktail for iPSC-CMs, resulting in increased force generation, cell area, and fatty-acid utilization [158].

### 5.7. In Vivo Maturation

hiPSC-CMs could mature extensively when transplanted in vivo, compared to in vitro cultures. After transplantation into healthy neonatal rat hearts, hiPSC-CMs exhibited an adult-like phenotype in structure, function, and gene expression profile within 2 months, indicating that the maturation is accelerated in a noncell-autonomous manner [161]. hiPSC-CMs can also achieve further maturation in a fast way even when transplanted into a diseased heart. hiPSC-CMs derived from an ACM patient were transplanted into neonatal animals and expressed more mature morphology after 1 month, including T-tubule formation, Cx43 expression, and calcium dynamics [162]. Although the mechanism for this accelerated in vivo maturation remains unknown, the natural environment with electromechanical stimulation, signaling through GJs, paracrine factors, and systemic factors could be the key factors [163].

Over time, considerable efforts have been devoted to improving the maturation of hiPSC-CMs, which show significantly improved genetic, morphological, and electrophysiological features. However, there are still issues remaining. Fully matured hiPSC-CMs similar to adult CMs have not been generated; it is still unclear whether patient-derived CMs could be matured in a manner similar to that of the normal hiPSC-CMs, while there are no methods to produce CMs with high maturation at a large scale and low cost.

**Table 3:** Strategies for enhancing hiPSC-CM maturation.

Strategy		Function	Signaling Pathway	Ref.
Long-term culture		Cellular hypertrophy, aligned sarcomere and M-bands	N.A.	[95,96]
Biochemical cues				
Hormone	T3, GC	Accelerate contractile force and metabolism maturation, T-tubules, cell-cycle arrest	PPARα/PGC1-α ↑	[97,98,99,100,101]
	Angiotensin II	Hypertrophy	MAPK ↑	[102]
Cytokine factor	IGF, NRG1	Growth and differentiation, hypertrophy, polynucleated, enhanced mitochondrial respiration	ERK ↑ PI3K-Akt ↑	[105,106,107]
	FGF, TGF-β, VEGF	GJ development, 3D structure modeling	MAPK ↑ PI3K-Akt ↑ TGF-β/Nodal pathway ↑	[108,109,110]
Others	Fatty acid	Mitobiogenesis, metabolic remodeling (metabolic switch from glycolysis to OXPHOS)	FAO/PPARα ↑ PI3K-AKT ↓	[111,112]
	O_2_	Accelerative differentiation, metabolic remodeling	HIF-1α/LDHA ↓ Wnt/β-catenin ↑	[113,115,116]
Physical cues				
Mechanical/electrical cues		Increased length/width ratio, rod-like morphology, aligned sarcomeres, improved ion activities, GJs polarization	Akt ↑ Ca^2+^/PKC/ERK ↑	[118,119,120,121]
ECM		Enhanced cellular organization, mitochondrial function and Ca^2+^ handling	p38 MAPK ↑ Akt ↑	[125,126,127]
Co-culture		Improved cell survival and cell size, aligned sarcomere, increased mitochondrial respiration; clear GJs and enhanced intercellular actions	AMPK ↑ cGMP–PKG ↑	[80,129]
3D culture		Profound maturation in all aspects, M-bands, developed tissue/organoid structure	MAPK ↑ Akt ↑	[134,135,136,137,138]
Regulation of gene expression				
Transcriptional level	Gene knockout or overexpression	Sarcomere development and mitochondrial respiration	SRF axis pathways↑ (such as HOPX, GATA, MEF2, PPARs/RXRs, ERRs)	[145,146,147,148,149,150,151,152,153,154]
Epigenetic modification	DNA methylation, histone modification, chromatin remodelling	Cell growth, sarcomere development, improved function	H3K27ac, H3K4me1, H3K4me3, and H3K9ac ↑	[89,156,157]
Posttranscriptional regulation	RNAi	Hypertrophy, α/β-MHC switching, aligned sarcomere, metabolic remodeling, improved Ca^2+^ handling	miR-1, let-7i, miR-125b-5p, miR-199a-5p, miR-221 and miR-222 ↑ miR-122 and miR-200a ↓	[158,159,160]
In Vivo Maturation		Adult-like phenotype with T-tubules, increased Cx43 expression and calcium dynamics	N.A.	[161,162]

T3, triiodothyronine; GC, glucocorticoid; IGF, insulin-like growth factor; NRG1, neuregulin-1; FGF, fibroblast growth factor; TGF-β, transforming growth factor beta; VEGF, vascular endothelial growth factor; OXPHOS, oxidative phosphorylation; GJ, gap junction; ECM, extracellular matrix; RNAi, RNA interference.

## 6. Functional Assessment of hiPSC-CMs for Drug Screening

As a powerful drug screening tool, the hiPSC-CM model must be used at the preclinical stage for new drug development while avoiding side effects. Three main cardiac functional parameters, electrical conduction, force generation, and cardiac ECC, have to be monitored for human heart drug discovery, and development of novel efficient assessment systems is still underway (Table 4) [19,164].

### 6.1. Electrophysiological Characterization

As a result of the opening and closing of ion channels located within the membrane, APs generate and propagate through the heart, and a series of ion channelopathies are related to the malfunction of ion channels [83]. Thus, ion channels have been widely studied for screening of drug targets, as well as for predicting drug side effects [165]. Functional assessment of electrophysiology is essential during drug screening of hiPSC-CM models.

The patch-clamp technique, regarded as the gold standard since its introduction, is a versatile electrophysiological tool for directly studying ion channel functions, and it opens a new avenue in drug discovery for ion channelopathies [166]. Although this method can offer accurate assessment of drug-induced arrhythmia at the cellular level, it is still considered to be low-throughput, invasive, and limited at the tissue level [167].

Microelectrode arrays (MEAs) are well accepted for recording the extracellular field potential (FP) of signal cardiac myocytes, as well as heart tissue, in a real-time, high-throughput, and noninvasive manner [168]. By recording the FP duration, the MEA system is able to measure both spontaneous and stimulated electrical activities, such as beat rate, electrical conduction velocity, refractory period, and AP length (QT interval), as extracellular FP is directly correlated with the intracellular AP [169]. Gilchrist et al. systematically assessed six arrhythmia-related parameters on the basis of an analysis of MEA recordings of hiPSC-CMs, and they discovered that the arrhythmia parameter variations detected were in agreement with clinical data when CMs are exposed to cardiotoxic drugs [170].

Although the multiparameter analysis system has been proven to be useful for arrhythmia drug screening, it is still considered as a cellular-level test for CMs dispersed in such culture systems. Stancesu et al. tested the electrical properties of hESC-CMs cultured in a patterned 2D system [171]. Such patterned MEA surfaces enabled hESC-CMs to be fabricated into tissues, which was not found in common MEA systems. However, a flat 2D culture system is not sufficient to produce a well-aligned sarcomere and originated GJs, essential for adult CM function. Li et al. developed a tissue-like cardiac construct on an MEA surface coated with aligned polydimethylglutarimide [172] or poly(lactic-*co*-glycolic acid) fibers [173]. Accordingly, hiPSC-CMs cultured on aligned fiber-coated MEA showed anisotropic propagation of the field potential and increased maturation, compared to 2D and random fiber coating culture systems, indicating that the 3D culture system is more reflective of in vivo systems.

### 6.2. Contractility

Contractile force and mechanical beating are more visual events for cardiac functional evaluation, especially in CMs with well-aligned sarcomeres. The deficiency in contractile force is directly responsible for some inherited and acquired heart diseases, such as DCM and HF [174,175]. The contraction and relaxation of CMs are consistent with the shift of myocardial fibers; thus, there is a predictable force–length relationship, named the Frank–Starling mechanism [176]. Furthermore, contractile force also responds to deformation of the ECM and, thus, can be detected in a direct or indirect manner.

Video–optical recording, usually containing a high-speed video microscope mounted with a motion vector analysis system, presents a noninvasive method for evaluation of contraction characteristics in beating cells [177]. Hayakawa et al. applied combined video–optical recording to drug response evaluation and investigated the relationship between contractile motion and electrophysiological changes in monolayer hiPSC-CMs [178]. Sala et al. employed video–optic measurements to a multisystem including monolayers, tissues and organoids, for quantifying cardiac muscle contractile force, both in vitro and in vivo [179].

Atomic force microscopy (AFM) has proven to be a versatile tool for the measurement of individual cellular or cluster mechanical properties such as not only beating and stiffness, but also Young’s modulus [180]. In the system, a scanning probe contacts the cells and senses mechanical signals that are later passed on to the mounted cantilever, causing cantilever deflection. Finally, cantilever deflections can be captured by an optical system [180]. Using the AFM method, Liu et al. described that iPSC-CMs generated from DCM patients showed decreased force and elasticity compared to normal samples at the cellular level [181]. According to this research, AFM can be applied to drug screening for subtle alternations in a dose-dependent manner. Furthermore, AFM is compatible with other detection methods such as MEA, as only synchronous recording of the beating force and electric events can explain the cardiac excitation–contraction coupling. Caluori et al. first implemented the combination of AFM/MEA with an in vitro DMD-derived iPSC-CM model, and stated that the method is sufficient as an assessment platform for the beating–force relationship of cells [182]. However, as neither the signal cell nor the cell cluster recapitulates the cell–cell organization in vivo or accounts for the cellular environment, none of the above studies illustrated the force–length relationship in vivo.

As discussed previously, researchers are focusing on 3D-EHTs because they can mimic the aligned and layered tissue structure of the native heart tissue. The muscle thin film (MTF) platform generated by Agarwal et al. is a higher-throughput tool at the tissue level, as the anisotropic iPSC-CMs cultured in soft cantilevers mimic the laminar architecture in vivo [183]. Upon muscle tissue contraction, the PDMS cantilevers deform proportionally to the force generated by the muscle tissue, and the deflection can be quantified using optical recording equipment, enabling contractility assessment. Their method first linked the cellular structure and function at the tissue level, and it proved to be a powerful tool for a functional assessment of drug screening and disease modeling. Wang et al. assembled BTHS iPSC-CMs and tested impaired contractile stress generation in a model using the MTF assay [70]. Garbern et al. applied a micromolded gelatin MTF to assess the functional maturity of generated iPSC-CMs [184]. In their research, both contractile force and beating decreased via downregulation of the mammalian target of rapamycin (mTOR) pathway, validating that the MTF assay is a functional platform for drug screening and disease mechanism studies.

### 6.3. Cardiac Excitation–Contraction Coupling (ECC)

Ca^2+^ dynamics bridge electrical excitation to contraction which is called ECC. The instantaneous changes in cytosolic calcium concentration during contraction and relaxation are referred to as calcium transient (CaT) [185]. It has been reported that the irregularity of CaT is one of the main causes of arrhythmia, as AP is induced by intercellular calcium [186,187]. Calcium indicator-based fluorescence imaging, also known as calcium mapping, is widely applied in cardiovascular drug evaluation [188,189]. By tracking intracellular Ca^2+^ flow, action potentials can be detected not only at the single-cell level, but also in the whole tissue, which adequately reflects the overall ECC.

Using individual hiPSC-CMs, Prajapati et al. performed simultaneous recordings of AP and CaT via patch clamp and calcium imaging, respectively, to further elucidate the mechanism linking calcium cycling and arrhythmias [190]. In their study, CaT was closely related to AP in both normal conditions and pathological conditions, such as EADs and DADs, revealing the complex dynamics and detailed mechanisms of arrhythmias. Moreover, using a CaT screening assay, Kopljar et al. developed an hiPSC-CM-based comprehensive risk quantification system in a simplified and high-throughput manner, providing a powerful tool to identify different arrhythmias, including torsades de pointes (TdP) [191]. As cytosolic calcium triggers cardiomyocyte contraction, CaT analysis is also regarded as complementary in the study of contractile force. Saleem et al. implemented a screening assay using hiPSC-CM EHTs for FFR research and confirmed a positive relationship between CaT and FFR but a reverse correlation between inotropic effect and frequency induced by omecamtiv mecarbil, a positive inotropic agent that strengthens heart performance [192]. Their work proved that a combination of calcium mapping and force monitoring is a reliable and effective method for routine drug testing.

**Table 4:** Assay methods for hiPSC-CM evaluation.

Assay Method	Parameter of CMs	Throughput	Invasive or Noninvasive	Specific Skill Required	Cost
Patch clamp	Membrane potential; ion channel current	Low	Invasive	Yes	Medium
Microelectrode array	Field potential	Medium	Noninvasive	No	High
Motion analysis [177]	Contractile force	Medium	Noninvasive	No	Low
Atomic force microscopy [181]	Contractile force; cell stiffness	Low	Invasive	Yes	Low
EHT [133]/muscle thin film [183]	Contractile force	High	Noninvasive	No	Low
Imaging [190]	Calcium transient; membrane potential	High	Medium invasive	No	Low

## 7. Update from the International Consortium for Novel Drug Testing Paradigm

After the development of new drug candidates, they need to pass the safety test before entering the market. The present safety testing paradigm (S7B/E14) is only based on drug-induced hERG channel blockade and the prolongation of QT interval, rather than directly focusing on the prediction of lethal proarrhythmic risk. A significant number of drug candidates could influence hERG [193] and are often rejected for further clinical development. However, some of these rejected drugs are free of proarrhythmic effects in both nonclinical and clinical assays [194]. In order to develop a more precise assessment technique, the United States (US) Food and Drug Administration called up a consortium of members from regulators, industry, and academia, which reached the conclusion that a novel paradigm should be established to replace the current one. The new paradigm, named comprehensive in vitro proarrhythmia assay (CiPA), includes a nonclinical in vitro assay on hiPSC-CMs and in silico modeling. It aims to discover the electrophysiological mechanisms underlying the possible proarrhythmic effect for drug candidates and is expected to become a pharmacological safety screening tool for drug development [195]. Recently, the CiPA international validation study was carried out across multiple sites with 28 blinded compounds [196], demonstrating the overall utility of the MEA methodology and hiPSC-derived CMs. This study also investigated the variation in evaluation results among different sites, suggesting that the three predictors, arrhythmia events, delayed repolarization, and repolarization prolongation caused by drugs, are sufficient to evaluate the efficacy of drugs with necessary accuracy. The data could support the claim that the CiPA has more accurate prediction of arrhythmia risk than the present guideline (S7B/E14).

The Japanese National Institute of Health Sciences (NIHS) also brought experts from multiple fields to develop a new testing paradigm for predicting clinical proarrhythmia risk, called the Japan iPS cardiac safety assessment (JiCSA) [197,198]. JiCSA developed an evaluation system using an MEA [117], multiple hiPSC-CM cell lines, and a selection of 60 compounds with different torsade de pointes (TdP) risks. By using a two-dimensional map [199], the relative TdP score was given to each compound. The data obtained by JiCSA demonstrated predictability of proarrhythmia risk, which could be reproduced in two cell lines from different suppliers [200,201]. The compounds selected by JiCSA overlapped with all 28 compounds selected by CiPA. In addition, the JiCSA data demonstrated good correlation with the CiPA study despite the different analysis methodologies [198]. Both CiPA and JiCSA have demonstrated the capability of hiPSC-CMs to evaluate proarrhythmia risk, which could be one of the most important applications of hiPSC-CMs. Collaboration among multiple sites around the world has already shown the reproducibility and robustness of the evaluation system.

In addition to the electrophysiology used by JiCSA and CiPA, contractility has been recently suggested as an evaluation factor for predictive safety assessment, since the cardiovascular liability of drugs occurs commonly via altered function of the contractile myocardium [202]. A multinational consortium comprising four academic teams and two companies was established, and a blinded evaluation of 28 drugs was carried out on 3D EHTs. The data indicated that the contraction amplitude was a good predictor of inotropes in 3D EHTs. With refinement of the test conditions, the platform-cell accuracy could be increased to 93%, compared with in vivo animal models.

There is scope for advances in various aspects, including improved maturation, combined subtype of cardiac cells, personalized medicine, scale-up production of hiPSC-CMs, and multiple readout, which could improve the application of hiPSC-CMs in drug development in the future.

## 8. Conclusions

hiPSCs induced from healthy and diseased donors and differentiated CMs are able to recapitulate the molecular and functional characteristics of the human heart. A large number of cardiomyopathies have been modeled, providing helpful tools for better understanding the cause of disease and for developing effective therapies. Since differentiated CMs tend to have less maturation than adult CMs, many efforts have been made for the functional and morphological improvement of hiPSC-CMs. Multiple readout methods, such as MEA, elastic pillar, motion analysis, and voltage dye, have been used for evaluating hiPSC-CMs. Novel drug testing paradigms (CiPA and JiCSA) have been proposed for better prediction of the proarrhythmic risk for drug candidates. By applying 28 or more standard drugs to the 2D monolayer hiPSC-CM tissue, these multisite collaborators have proven the capability of hiPSC-CMs to evaluate proarrhythmia risk. In the future, by integrating more maturation and multiple readout technologies into the new paradigms, the robustness and accuracy can be further improved. Similarly, patient-specific iPSC-CMs could help develop personalized therapy.

## Figures and Tables

**Table 2:** Main characteristics of hiPSC-CMs and human adult CMs.

Characteristics	Parameters		hiPSC-CMs	Human Adult CMs	Assessment Methods
Morphology and Microstructure	Cell Shape		Round shape	Rod shape, anisotropic	Imaging Immunostaining to assess structural features
	Cell size	Length	5–10 μm (diameter)	150 μm	
		Width		20 μm	
		Height	5 μm	15 μm	
		Volume	2000 μm^3^	40,000 μm^3^	
	Length/width ratio		—	7:1	
	Nucleation and ploidy		Mononucleated, diploidy	Binucleated (25%) and polyploidy	
	Sarcomere		1.6 μm, disorganized	1.8 μm (contracted)-2.2 μm (relaxed), organized	
	Enriched isoforms		α-MHC, ssTnI, MLC2A, N2BA, SMA	β-MHC, cTnI, MLC2V, N2B	
	Other microstructures		Lack T-tubules and M-band; poor SR, mitochondria; circumferential IDs	Developed and abundant microstructures; polarized IDs	
Electrophysiology	Beating		Beating spontaneously or stimulated by a 0.08–4 mN/mm^2^ force	Beating only when stimulated by a 40–80 mN/mm^2^ force	Patch clamp and MEAs for ion channels and AP currents Video-optical recording, atomic force microscopy (AFM). and muscle thin films (MTFs) for contractile force measurements (Frank–Starling relationship)
	Membrane capacitance		~20 pF	~190 pF	
	Conduction velocity		10–20 cm/s	60 cm/s	
	Upstroke velocity		10–50 V/s	150–350 V/s	
	Action potential		−60 mV (like nodal)	−90 mV	
	Specific currents		*I*_Kf_	*I*_K1_	
Calcium Handling	ECC		Slow	Fast	Calcium imaging using fluorescent calcium indicators
	Ion channels		NCX	LTCC-β2 (20-fold higher), RyRs (1000 folds higher), calsequestrin, SERCA	
Metabolism	Mitochondria		Round shape with poor cristae	Oval shape with developed cristae; active fission and fusion	Mass spectrometry (MS) and nuclear magnetic resonance spectroscopy (NMR) for metabolic flux assays Oxygen consumption and extracellular acidification rate to access mitochondria respiration Imaging and fluorescent staining for mitochondrial membrane potential (MMP)
	Abundance (% to cell volume)		<5%	~30%	
	Location		Perinuclear space	Between myofibrils and under sarcolemma	
	Metabolic substrate		Glucose (85%), fatty acid (15%)	Fatty acid (80%), glucose (20%)	
	ATP source		Anaerobic glycolysis	FAO	
Gene Expression	Upregulated genes		Cell-cycle genes: *CDK* Automaticity genes: *HCN4*, *KCNJ2* Fetal/natal isoform genes Glycolysis-related genes	Cell-cycle arrest genes: *CDKI* Overall upregulation of structure organization and function development genes	Imaging Flow cytometry to access cell cycle Fluorescent staining

AFM, atomic force microscopy; AP, action potential; cTnI, cardiac muscle troponin I; ECC, excitation–contraction coupling; FAO, fatty-acid oxidation; ID, intercalated disc; LTCC-β2: L-type calcium channel β subunit; MEA, microelectrode array; MLC2A, myosin regulatory light chain 2 atrial isoform; MLC2V, myosin regulatory light chain 2 ventricular isoform; MMP, mitochondrial membrane potential; MS, mass spectrometry; MTF, muscle thin film; N2B; titin isoform type containing only N2B elements; N2BA, titin isoform type containing both N2A and N2B elements; NCX, Na^+^–Ca^2+^ exchanger; NMR, nuclear magnetic resonance spectroscopy; RyR2, ryanodine receptor 2; SERCA, sarco/endoplasmic reticulum Ca^2+^ ATPase; SMA, smooth muscle actin; SR, sarcoplasmic reticulum; ssTnI, slow skeletal muscle troponin I; α-MHC, myosin heavy chain α-isoform; β-MHC, myosin heavy chain β-isoform; pF, picofarad.

## Abbreviations

ACM	Arrhythmogenic cardiomyopathy
AFM	Atomic force microscopy
AJ	Adhesive junction
AP	Action potential
APD	Action potential duration
ARVC	Arrhythmogenic right-ventricular cardiomyopathy
BTHS	Barth syndrome
CaT	Calcium transient
CFCS	Cardio-facio-cutaneous syndrome
CHF	Chronic heart failure
CiPA	Comprehensive in vitro proarrhythmia assay
CM	Cardiomyocyte
CPVT	Catecholaminergic polymorphic ventricular tachycardia
CVD	Cardiovascular disease
DAD	Delayed afterdepolarization
DCM	Dilated cardiomyopathy
DMD	Duchenne muscular dystrophy
EAD	Early afterdepolarization
EC	Endothelial cell
ECC	Excitation–contraction coupling
ECG	Electrocardiogram
ECM	Extracellular matrix
EHT/EHM	Engineered heart tissue/engineered heart muscle
ER	Endoplasmic reticulum
ERR	Estrogen-related receptors (including α, β, and γ)
FAO	Fatty-acid oxidation
FFR	Force–frequency relationship
FGF	Fibroblast growth factor
FP	Field potential
GAA	Acid-α-glucosidase
GATA	GATA family transcription factor
GC	Glucocorticoid
GJ	Gap junction
HCM	Hypertrophic cardiomyopathy
HCN4	Hyperpolarization-activated cyclic nucleotide-gated potassium channel 4
hERG	Human ether-à-go-go-related gene
HF	Heart failure
HFrEF	Heart failure with reduced ejection fraction
HIF1	Hypoxia-inducible factor 1-alpha
hESC	Human embryonic stem cell
hiPSC	Human induced pluripotent stem cell
hiPSC-CM	Human induced pluripotent stem cell-derived cardiomyocyte
HOPX	Homeodomain-only protein homeobox
ID	Intercalated disc
IGF	Insulin-like growth factor
JiCSA	Japan iPS cardiac safety assessment
LDHA	Lactate dehydrogenase A
LQT1/2/3	Long QT syndrome type 1/type 2/type 3
LQTS	Long QT syndrome
LTCC	L-type calcium channel
MEA	Multielectrode array
MEF2	Myocyte enhancer factor-2
miRNA/miR	MicroRNA
MMP	Mitochondrial membrane potential
MS	Mass spectrometry
MSC	Mesenchymal stem cell
MTF	Muscle thin film
NCX	Na+–Ca2+ exchanger
NMR	Nuclear magnetic resonance spectroscopy
Non-CM	Noncardiomyocyte
NRG1	Neuregulin-1
NRs	Nuclear receptor superfamily
NT-proBNP	N-terminal pro B-type natriuretic peptide
OXPHOS	Oxidative phosphorylation
PD	Pompe disease
pF	Picofarad
PGC-1	Peroxisome proliferator-activated receptor-γ co-activator 1-alpha/beta
PPAR	Peroxisome proliferator-activated receptor
RA	Retinoic acid
RXR	Retinoid X receptor
RyR	Ryanodine receptor
SERCA	Sarco/endoplasmic reticulum Ca2+ ATPase
SR	Sarcoplasmic reticulum
SRF	Serum response factor
ssTnI	Slow skeletal muscle troponin I
T3	Triiodothyronine
TdP	Torsade de pointes
TGF-β	Transforming growth factor beta
TNNT	Troponin
TTN	Titin
T-tubule	Transverse tubule
VEGF	Vascular endothelial growth factor
α-MHC	Myosin heavy chain α-isoform
β-MHC	Myosin heavy chain β-isoform
